# Multi-Fractal Hierarchy of Single-Walled Carbon Nanotube Hydrophobic Coatings

**DOI:** 10.1038/srep08583

**Published:** 2015-02-26

**Authors:** Francesco De Nicola, Paola Castrucci, Manuela Scarselli, Francesca Nanni, Ilaria Cacciotti, Maurizio De Crescenzi

**Affiliations:** 1 Dipartimento di Fisica, Universitá di Roma Tor Vergata, Via della Ricerca Scientifica 1, 00133 Roma, Italy; 2 Istituto Nazionale di Fisica Nucleare, Universitá di Roma Tor Vergata (INFN-Roma Tor Vergata), Via della Ricerca Scientifica 1, 00133 Roma, Italy; 3 Dipartimento di Ingegneria dell'Impresa, Universitá di Roma Tor Vergata (INSTM-UdR Roma Tor Vergata), Via del Politecnico 1, 00133 Roma, Italy; 4 Universitá di Roma Niccoló Cusano (INSTM-UdR), Via Don Carlo Gnocchi 3, 00166 Roma, Italy; 5 Istituto di Struttura della Materia, Consiglio Nazionale delle Ricerche (ISM-CNR), Via del Fosso del Cavaliere 100, 00100 Roma, Italy

## Abstract

A hierarchical structure is an assembly with a multi-scale morphology and with a large and accessible surface area. Recent advances in nanomaterial science have made increasingly possible the design of hierarchical surfaces with specific and tunable properties. Here, we report the fractal analysis of hierarchical single-walled carbon nanotube (SWCNT) films realized by a simple, rapid, reproducible, and inexpensive filtration process from an aqueous dispersion, then deposited by drytransfer printing method on several substrates, at room temperature. Furthermore, by varying the thickness of carbon nanotube random networks, it is possible tailoring their wettability due to capillary phenomena in the porous films. Moreover, in order to describe the wetting properties of such surfaces, we introduce a two-dimensional extension of the Wenzel-Cassie-Baxter theory. The hierarchical surface roughness of SWCNT coatings coupled with their exceptional and tunable optical and electrical properties provide an ideal hydrophobic composite surface for a new class of optoelectronic and nanofluidic devices.

The hydrophobicity of solid surfaces is an important property in a variety of natural^1^,^2^ and technological processes^2^,^3^,^4^,^5^,^6^ with several industrial applications such as waterproof surfaces^7^, anti-sticking^8^, anti-contamination^9^, self-cleaning^6^, anti-fouling^10^, anti-fogging^11^, low-friction coatings^12^, adsorption^13^, lubrication^14^, dispersion^15^, and self-assembling^5^.

Generally, the realization of artificial hydrophobic surfaces relies on two main features: the surface material chemical composition and its morphological structure. Usually, the chemical composition is an intrinsic property of materials but it can be controlled^7^,^16^ to lower material surface tension. On the other hand, micro- and nano-morphology (surface roughness^1^,^17^) may also be enhanced especially by exploiting hierarchical^1^,^2^,^9^,^12^,^18^ and fractal architectures^3^, possibly allowing air pocket formation to further repel water penetration^19^. Nevertheless, realizing a permanent hydrophobic surface remains quite a challenge. Recently, time durability^20^, chemical^21^, mechanical^12^, and thermal stability^22^ have been addressed. Among the numerous materials satisfying the two aforementioned conditions, carbon nanotubes^23^ offer versatility, stability, and multi-functionality owing to their exceptionally unique properties^24^, making their usage widespread in hydrophobic surface realizations^3^,^7^,^8^,^9^,^12^,^13^,^21^,^22^,^25^,^26^,^27^,^28^,^29^,^30^,^31^.

Single-walled carbon nanotubes have the *sp*^2^ lattice of graphitic carbon, thus they are inherently hydrophilic (graphite contact angle ≈ 86°^14^) but apolar. However, surface functionalization or textured arrangement may facilitate the easy control of their wetting properties^3^,^9^,^29^. Moreover, SWCNTs have unique optical and electrical properties with both metal and semiconducting features^24^. Therefore, understanding their hydrophobic properties is crucial to realize conductive, optically active, and hydrophobic coatings paving the way to a new generation of optoelectronic and nanofluidic devices.

Nevertheless, the multi-step fabrication of carbon nanotube films involving advanced processes is cost inefficient, laborious, substrate selective, and time consuming, posing challenges to versatility and large scale production of hydrophobic carbon nanotube coatings. However, self-assembly hierarchical nanostructured materials such as carbon nanotube films^32^,^33^ are nowadays investigated as a consequence of their tunable peculiar properties, easy, high reproducible, and low-cost fabrication. In addition, they are ideal low-dimensional materials for the fabrication of high aspect ratio and large area devices. Despite surface hierarchy relevance, there is a lack of consensus on how to incorporate its description into physical models.

In this article, we report SWCNT films (Figure 1a) prepared by a simple, rapid and inexpensive vacuum filtration process of a SWCNT aqueous dispersion. We then investigated the wettability of our films depositing them at room temperature, by the dry-transfer printing method^27^,^34^,^35^ on glass (Figure 1b), silicon (Figure 1c), and plastic foils (Figure 1d). Differently from other authors our fabrication protocol of SWCNT films (see Methods) does not involve complex industrial processes such as chemical vapor deposition. Therefore, our dry-transfer printing technique is practicable also from liquid dispersions of carbon nanotubes. Moreover, simply by varying the film thickness it is possible tailoring SWCNT wettability in a controlled fashion. Once characterized our samples, we will detail the multi-fractal nature of our films in order to quantify their hierarchical surface morphology. We will reserve the second part of the article to the discussion of the observed results suggesting a two-dimensional extension of the Wenzel^17^ and Cassie-Baxter^19^ theories, to include the film hierarchical morphology in the surface wetting model. Finally, capillary phenomena in SWCNT porous networks will be discussed.

## Results

### Sample characterization

**Figure 2:**
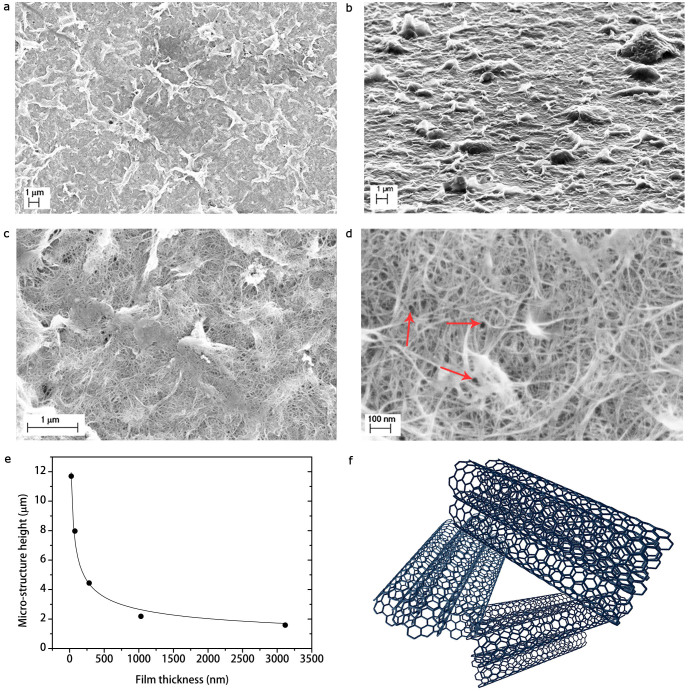
Investigation of the SWCNT film morphology. Scanning electron micrographs of a SWCNT film at different magnifications 10,000× (a,b), 50,000× (c), and 200,000× (d). (b), In the image taken at grazing angle (≈ 90° respect with the sample plane normal), it is possible to observe that micro-structures consist of self-assembly ripples made of surfactant and SWCNTs, as evident in (c). (d), Pores in the SWCNT network (marked with red arrows). (e), Micro-structure height as a function of film thickness. (f), Three-dimensional sketch of a pore in the SWCNT network.

In Figure 2a–d scanning electron microscopy (SEM) images taken at different magnifications of the SWCNT films obtained from the process described in Methods are reported. At lower magnification (Figure 2a), the films appear corrugated with asperities or ripples characterized by the lighter contrast of the image. The presence of such a roughness randomly distributed on the film surface is further confirmed by SEM images acquired at grazing angle respect to the sample surface (Figure 2b). It is worth noting that such a morphology is typical of every our SWCNT films independently on the substrate underneath. Furthermore, we obtained statistical values for the ripple areas 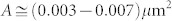, by analyzing SEM images at a magnification of 50,000× (Figure 2c). We remark that the dispersion of ripple area values arises from the large variety of structures, which naturally occurred in the film. We also determined ripple heights 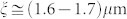 from SEM micrographs acquired at grazing incidence, by calculating the projection along the film surface normal of the measured heights at given incidence angle. Interestingly, we found that ripple height values decrease as the film thickness increases (Figure 2e). Furthermore, the achieved values in the micrometer range for the ripple height allow us to refer in the following to them as the SWCNT film micro-structures.

Moreover, at higher magnifications (Figure 2c,d), SEM micrographs reveal that the whole film surface, including the aforementioned micro-structures are made of a porous random network of self-assembly elongated structures with diameters 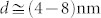. Such diameter values suggest that SWCNTs are aggregated in small bundles, since they are expected to have diameters in the range ≈ (0.7–1.0) nm (see Methods). Therefore, hereafter we will refer to the SWCNT bundles as the film nano-structures. Consequently, since the observed self-assembly micro-structures in the film are made of nano-stuctured SWCNT bundles, the SWCNT film morphology can be reasonably considered as a two-fold hierarchy.

In addition, we measured the SWCNT network pore area, *i.e.* the area of the irregular, empty regions delimited by the intersection among carbon nanotube bundles (marked with red arrows in Figure 2d and sketched in Figure 2f). This allows us to estimate the value of the pore radius 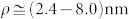 of the SWCNT network, defined as the radius value of the circle equal to the pore irregular area. It is worth noting that all the previous experimental values have been estimated by taking the average of the mode value distribution obtained for each sample. This operation is reasonable, since results obtained from each SWCNT film are independent on the specific film; therefore our samples are statistically similar.

Moreover, we ascribe the occurring of self-assembly micro-structures in the SWCNT film to a bending process during evaporative drying^32^,^33^ of the film throughout its preparation (see Supplementary Section I for the detailed analysis). In our proposed mechanism, the dry-induced out-of-plane micro-structure assembly is the result of the competition between capillary force in the SWCNT porous network and bending stress due to the elasticity of SWCNT films. Indeed, according to the Kirchoff-Love plate theory^36^, it can be shown that the maximum mid-surface out-of-plane radial deection of the circular SWCNT thin film with radius *r*, bending stiffness Δ, and capillary force *F_cap_* is given at first approximation by 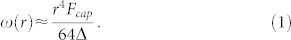
The value of this quantity is compatible with the experimental micro-structure height *ξ* of the SWCNT films. Furthermore, since Δ ∝ *τ*^3^ (see Supplementary Section I) we confirmed the observation of the decrease of *ξ* with the increase of film thickness *τ*, as shown in Figure 2e. Once the water used during the rinsing step of the SWCNT film fabrication is completely evaporated, a pattern of micrometer-sized randomly shaped islands is formed. If after complete evaporation there is a balance between attractive capillary and elastic energies, the nano-structures are in a stable bent configuration, with respect to further wetting-dewetting cycles^37^. Similar hierarchical structures have been reported also for relatively soft materials such as ceramic glazes^38^, clay soils^39^, and metal oxides^37^. The reported self-assembly leads to a hierarchical micro- and nano-structured roughness, as those found in lotus leaves^2^ and rose petals^1^, which may enhance SWCNT film hydrophobic properties.

**Figure 3:**
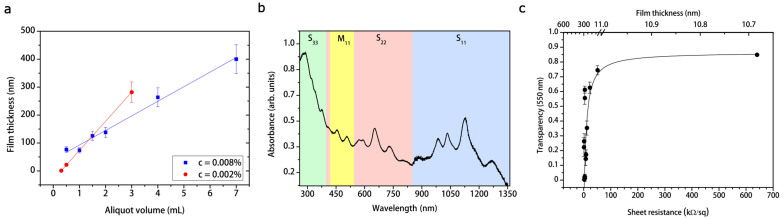
Characterization of the SWCNT film. (a), Thickness of SWCNT films as a function of aliquot volume of the SWCNT dispersion. The film thickness varies also as a function of different dispersion concentrations. We report for instance, thickness values for c = 0.008% (blue squares) and *c* = 0.002% (red dots). (b), Absorption spectrum of a SWCNT film. Colored regions represent the first *S*_11_, the second *S*_22_, and the third *S*_33_ optical transition range for semiconducting chirality and the first *M*_11_ range for metallic chirality. (c), Transmittance at 550 nm as a function of the SWCNT film sheet resistance and thickness. The ticker the film, the lower the transmittance, therefore the lower the sheet resistance. Uncertain bars represent systematic errors.

Furthermore, we investigated our SWCNT coating optical and electrical properties. Optical spectroscopy was performed to estimate the SWCNT film thickness *τ* through the Lambert-Beer law *A*(*λ*) = *α*(*λ*)*τ*, where *A*(*λ*) and *α*(*λ*) are the absorbance and the absorption coefficient at a given wavelength *λ*, respectively. For instance, we used the value *α*(550) = (6.2 ± 0.8) *µ*m^−1^ obtained from optical and X-ray photoemission spectroscopy (XPS) measurements^40^. We found that there is a linear dependence between the thickness of the obtained SWCNT film and the aliquot volume *V* of the same dispersion (Figure 3a). Since *V* = *m*/*c*, where *m* is the SWCNT mass in the aliquot and *c* the concentration of the dispersion, the SWCNT film thickness can be controlled suitably varying aliquot volume and/or dispersion concentration. As plotted in Figure 3a, we found the empirical law *τ* = *C*_1_ + *C*_2_*V* for two different dispersion concentrations (*c* = 0.008%, *c* = 0.002%), where *C*_1_, *C*_2_ are two constants dependent on the SWCNT dispersion preparation (*i.e.* SWCNT and surfactant concentrations). Therefore, our film fabrication method allows us to easily control the SWCNT film thickness.

In Figure 3b, we can observe the SWCNT film optical spectrum throughout the UV/Vis/NIR range. We may relate absorption peaks to different optical transitions of semiconducting (*S_ii_*) and metallic (*M*_11_) SWCNTs of different chirality^24^,^41^. Therefore, we infer that the as-realized films consist of mostly semiconducting (≈ 90% in percentage) SWCNTs with a broadband absorption.

As fitted in Figure 3c, the variation of the transmittance *T* calculated for instance at 550 nm of SWCNT films with their sheet resistance *R_s_* follows the relation^42^

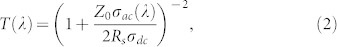
where *Z*_0_ = 377 Ω is the free-space impedance and *σ_dc_*, *σ_ac_*(*λ*) are respectively the electrical and optical conductivity. Since the sheet resistance is related to the film thickness *τ* by *R_s_* = (*σ_dc_τ*)^−1^, the thicker the film, the lower the sheet resistance, therefore the transparency values. Our SWCNT coatings may have low sheet resistance values with high transparency, for instance a film ≈ 77 nm thick has a transparency ≈ 74% and a sheet resistance ≈ 50.8 kΩ *sq*^−1^. We remark that the percentage of metallic nanotubes is the same for films with different thickness values, therefore the observed change in sheet resistance is independent on the percentage of metallic chirality.

### Contact angle measurements

**Figure 4:**
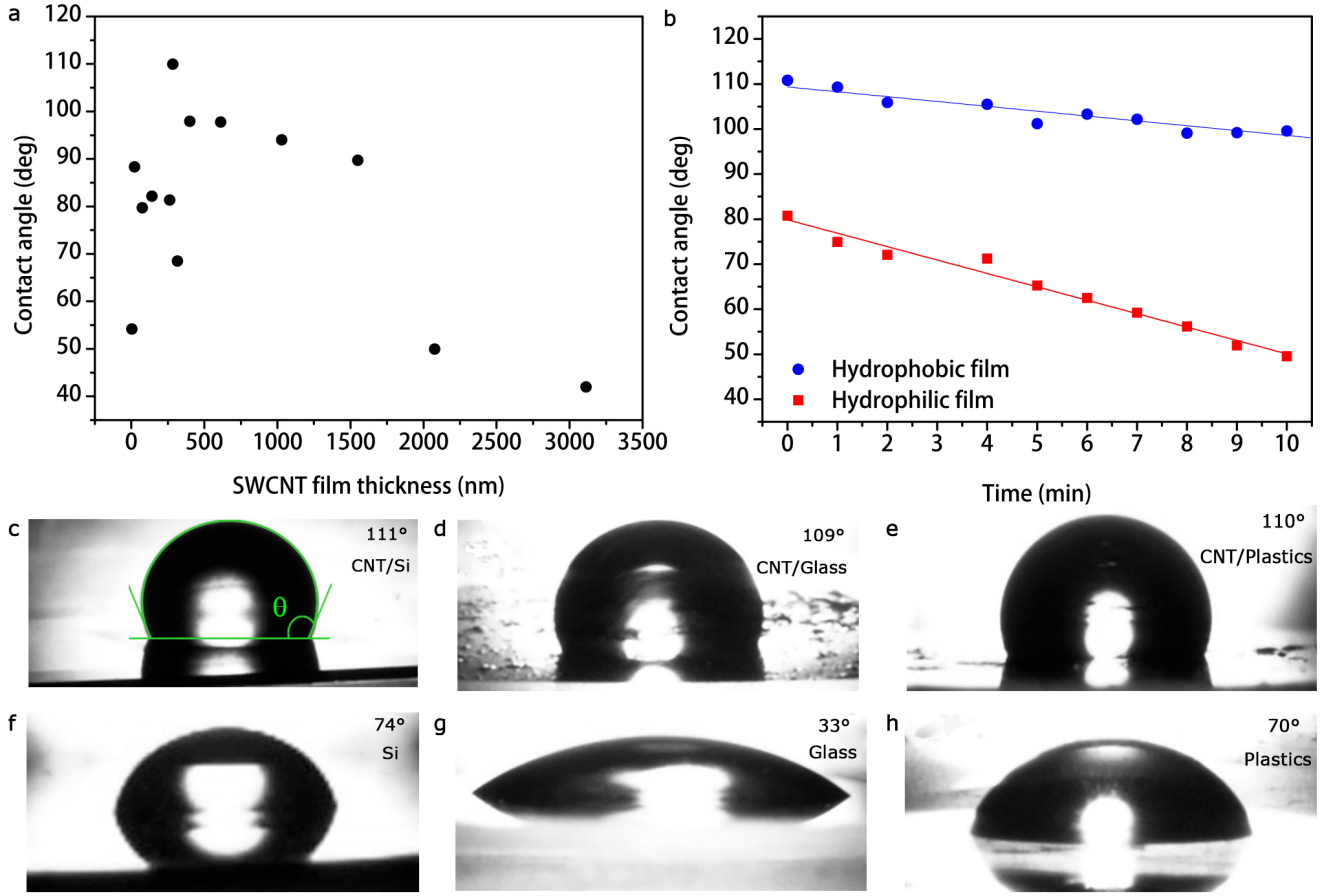
Contact angle measurements. (a), Contact angle as a function of SWCNT film thickness. (b), Water drop contact angle for hydrophilic (red squares) and hydrophobic (blue dots) SWCNT films as a function of elapsed time after drop cast. The linear decrease of *θ*(*t*) in time is due to suction by the porous film and not only to evaporation of liquid, otherwise the contact angle would be constant in time but not the droplet volume. Water droplets on SWCNT films deposited on silicon (c), glass (d), plastics (e), and on bare silicon (f), glass (g), and plastics (h).

In Figure 4a and in Table 1 the experimental water contact angle values (see Methods) for SWCNT films deposited on glass slides are reported as a function of film thickness. In particular, data show that we can control the wetting properties of SWCNT films by varying their thickness. Actually, films ≈ 282–1030 nm thick result to be hydrophobic (*θ* > 90°), otherwise hydrophilic. Interestingly, sample wetting properties appear not to be influenced by the substrate. As shown in Figure 4c–e, several hydrophilic substrates (see Table 1) such as silicon (Figure 4f), glass (Figure 4g), and plastic foils (Figure 4h) resemble similar water droplet profiles once covered by the hydrophobic SWCNT film with the thickness value corresponding to the highest contact angle reported (≈ 110°). Therefore, semitrans-parent thin SWCNT films may be hydrophobic and electrically conductive coatings for hydrophilic and insulating or semiconducting substrates, achieving contact angle values comparable to polydimethylsiloxane (PDMS), which indeed exhibits ≈ 110°.

Moreover, it was not possible measuring the contact angle (*θ* ≈ 0°) of acetone, ethanol, or glycerol because SWCNT films were totally wet by those liquids. Hence, our SWCNT films are super-lipophilic (*θ* < 5°) as the consequence of their hydrophobic and apolar behavior.

In order to investigate the porosity of SWCNT films, we also performed suction experiments. Figure 4b reports the variation of the contact angle *θ*(*t*) as a function of the elapsed time from drop cast on the coating, for a hydrophobic (≈ 109°) and hydrophilic (≈ 81°) SWCNT film deposited on a glass substrate. The rapid linear decrease of the contact angle in time is not only due to liquid evaporation (otherwise the contact angle would be constant in time but not the droplet volume *V*), but also to suction following the law^15^
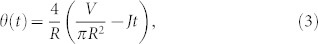
From linear fits in Figure 4b, we extracted the drop radii *R*_1_ = (0.488 ± 0.001) mm, *R*_2_ = (0.542 ± 0.002) mm and the hydrodynamic flows *J*_1_ = (15 ± 2) *µ*L cm^−2^ s^−1^, *J*_2_ = (40 ± 2) *µ*L cm^−2^ s^−1^ of the water through the SWCNT film, respectively for the hydriphobic and hydrophilic SWCNT films. The fits confirm that for a hydrophobic coating the flow is slower than for a hydrophilic film. However, in the limit of super-hydrophobicity the hydrodynamic flow is null and the hydrophobic state is said to be durable in time.

### Fractal analysis of carbon nanotube random network films

Fractal geometry has attracted wide attention as a tool to describe real surfaces, in particular in solid physics^4^,^43^,^44^,^45^,^46^,^47^,^48^. Indeed, the fractal nature of fracture or network surfaces of real solids has been confirmed by numerous experimental studies^49^,^50^. The recent introduction of fractal analysis as a refinement on the conventional roughness parameters provides a powerful method capable of revealing systematic differences in textured or rough surfaces. Generally, not only it is a hard task estimating the surface asperity dimensions, especially when the surface has a very complex and variable morphology, but also this measurement suffers of a huge systematic error, due to the approximations made by using the Euclidean measure to merely quantify the surface asperity size.

A fractal surface is a geometrical object with a fractal dimension 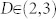, which has a non-integer value. In general, a fractal surface has two main properties: scale-invariance and self-similarity. The former, valid in a statistical sense follows from the scaling law^47^ *m*(*σs*) = *m*(*s*)*σ^D^*^−2^, where *m*(*σs*) is the scaled measure of the fractal length *s*, *m*(*s*) is the measure of the fractal, and *σ* is the scaling factor. Evidently, the fractal dimension does not change under coordinate transformations. On the other hand, self-similarity derives from the scale-invariance property, meaning that every part of a fractal surface is equal to the whole surface. Moreover, in real fractal surfaces, since the fractal dimension is local, it is possible that *D* is not uniquely defined and may vary from region to region on the surface, depending on surface complexity. Therefore, there is a distribution of several fractal dimensions with a mean value reecting a multi-fractal behavior.

Scanning electron microscopy has revealed itself to be a reliable instrument for fractal analysis^46^, comparable to other scanning probe microscopy such as atomic force microscopy (AFM)^51^, scanning tunneling microscopy (STM)^43^, and profilometry^44^. In particular, the SEM image of a fractal surface is at most self-affine (self-similar not in every spatial directions), because the obtained two-dimensional topographic image has no height profile. This does not represent a big drawback, since self-affinity just leads to a little underestimation of the real fractal dimension of the surface^44^. Nevertheless, the evaluation of *D* through the fractal analysis of SEM images has three main advantages. The first one lies in the high resolution of the image (typically 1024 × 768 pixels) which allows a high density of pixels. Secondly, the possibility of acquiring images at scales spanning many orders of magnitude allows to investigate if the surface structure varies over a large size range. Thirdly, in SEM images there is no convolution of the probe tip, which typically introduce a systematic error in the estimation of the fractal dimension of the surface^48^.

There are several techniques for practical estimation of the real surface fractal dimension, such as the box counting method^43^, the triangulation algorithm^43^, the variogram^46^, and the Fourier power spectrum analysis^43^. We established the validity of all those algorithms testing them on deterministic fractals with known fractal dimensions and finding that the triangulation method is the most reliable and sensitive to multi-fractality (see Supplementary Section II).

**Figure 5:**
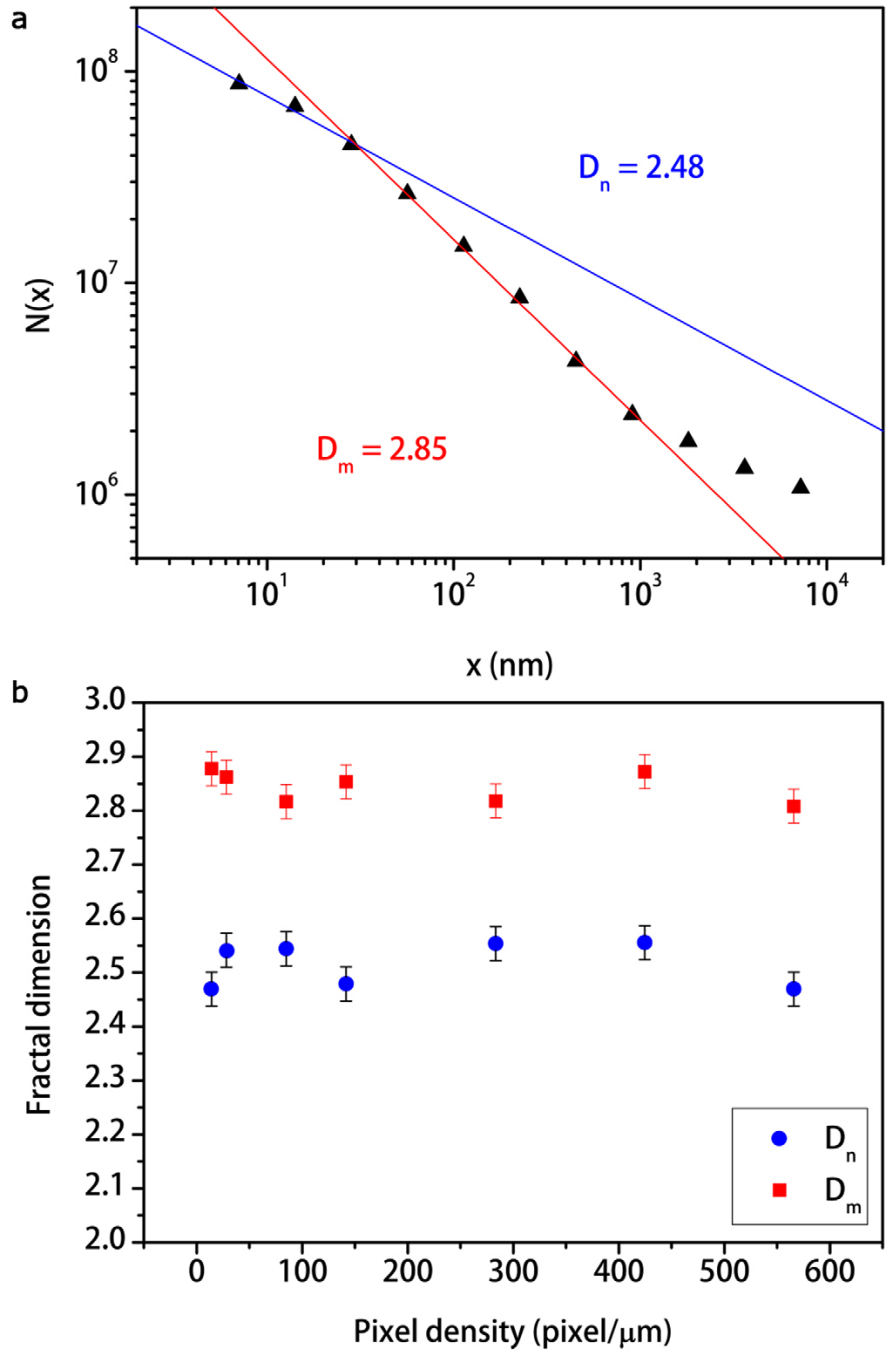
Fractal analysis of a SWCNT film by triangulation method. (a), The log *N*(*x*)-log *x* plot exhibits two fractal dimensions *D_m_* = 2.85 ± 0.03, *D_n_* = 2.44 ± 0.03 with maxima 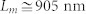, 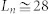 and minima 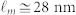, 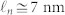 fractal lengths, for the sample 1 SWCNT in Table 1. High values of the triangle size *x* beyond *L_m_* value correspond to the edge of the SEM image. Therefore, the number of all triangles that contain at least one pixel of the image *N*(*x*) is close to zero returning *D* = 2 as dimension of the plane. (b), The graph shows that fractal dimensions *D_m_* (red squares) and *D_n_* (blue dots) do not change with SEM image pixel density, demonstrating thereby the scale-invariance and self-affinity properties. The estimation of the systematic error on *D* is the average of the semi-dispersion between the known theoretical values of the test fractals and the measured values, over all the tested fractals.

In order to measure the fractal dimensions for our samples, we chose an image magnification of 50,000× corresponding to a density of pixels of 141 pixel/*µ*m in which both micro- and nano-morphology are present (Figure 2b). In Table 1 the measured values of fractal dimensions for the as-realized SWCNT films are reported. Interestingly, the triangulation method (see Methods) distinguishes two main fractal dimensions *D_m_*, *D_n_* that we may respectively attribute to the SWCNT film micro- and nano-structures (Figure 5a). Furthermore, these values are in agreement with those of fracture surfaces and fault patterns for heterogeneous natural fractals such as rocks^47^ and seismic waves^50^. Moreover, all samples have similar fractal dimensions with average values over all samples *D_m_* = 2.83 ± 0.01, *D_n_* = 2.46 ± 0.01 with maxima 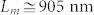, 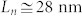 and minima 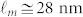, 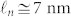 fractal lengths. Fractal length represents the correlation length within the fractal scaling law holds. The maxima and minima fractal lengths may be respectively considered as upper and lower limits of the micro- and nano-structure sizes. However, the fact that each fractal dimension is quite the same for every sample confirms the universality^50^ of the fractal dimension for random networks and it is a direct consequence of the SWCNT film randomness and heterogeneity. We also performed the same fractal analysis at different scales of the SEM images for a given sample. In Figure 5b we observe that fractal dimensions are quite constant as a function of the pixel density, thereby demonstrating scale-invariance and self-affinity properties of SWCNT random networks, despite of the large variety of morphological features in the film with different Euclidean dimensions.

These results are particularly interesting, since our SWCNT films exhibit similar morphological features and fractal behavior in statistical sense, for different SWCNT dispersion concentrations and filtration speeds. In particular, samples 6, 9, 11 in Table 1 have been obtained with *c* = 0.002%, while for the other samples *c* = 0.008%. Moreover, it was not possible keeping the filtration speed constant, because the thicker the film, the denser the film, therefore the slower the filtration speed. We can thus assert that the above fractal analysis is independent both on film thickness and dispersion concentration.

## Discussion

### Two-dimensional wettability model for carbon nanotube hierarchical fractal surfaces

The contact angle *θ* between a chemically homogeneous smooth solid surface and a liquid droplet obeys to the Young's equation^15^

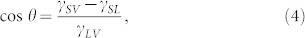
where *γ_SV_*, *γ_SL_*, and *γ_LV_* denote surface tensions of the solid-vapor, the solid-liquid, and the liquid-vapor interfaces, respectively. On the other hand, for a chemically homogeneous rough surface the experimental contact angle θ*, often referred as the apparent contact angle is described by the Wenzel's law^17^

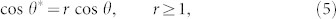
where *θ* is the Young's contact angle and *r* is the roughness factor, which gives an estimation of the effective solid surface contact area wet by liquid. The roughness factor indeed takes into account the morphology of the solid surface, as it is defined as the surface ratio between the rough surface and its corresponding geometrical projection on the plane^17^. In particular, for a fractal surface Li *et al.*^3^ showed that *r* can be expressed as 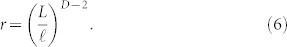
In Table 1 micro- (*r_m_*) and nano-structure (*r_n_*) roughness factors for each samples are reported.

**Figure 6:**
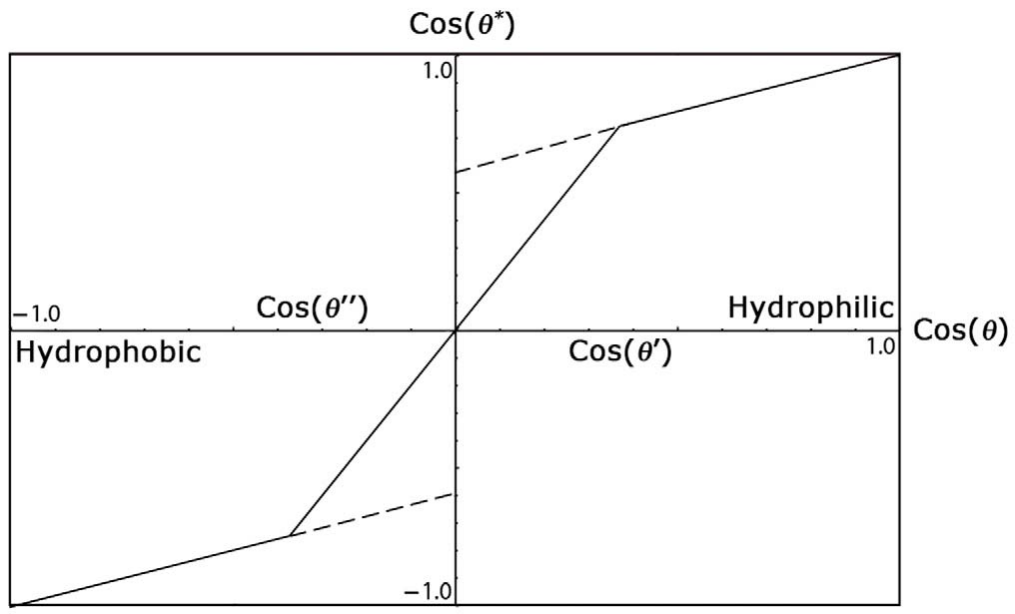
Wenzel-Cassie-Baxter one-dimensional phase diagram. The parameters used to compute the plot are for instance *r* = 2 and *ϕ* = 0.4. It is possible distinguishing four main states. The Cassie-Baxter partial wetting state (cos *θ*′ < cos *θ* < 1), the Wenzel partial wetting state (0 < cos *θ* < cos *θ*′), the Wenzel non-wetting state (cos *θ*″ < cos *θ* < 0), and the Cassie-Baxter non-wetting state (−1 < cos *θ* < cos *θ*″). In addition, there are metastable states (dashed lines) where both Wenzel and Cassie-Baxter states coexist.

Moreover, for a rough composite surface air or liquid may remain trapped in the surface asperities. Therefore, a fraction *ϕ* of the sample solid surface is directly in contact with the droplet total area, while the other fraction will first contact an air (*ϕ*_−_) or liquid (*ϕ*_+_) layer underneath the droplet. In this situation, the apparent contact angle is given by the one-dimensional Cassie-Baxter's equation^19^

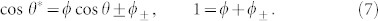
Equation (7) well describes the hydrophobic (−) and hydrophilic (+) states leading respectively to the air pocket and the precursor liquid film formation^15^. In Figure 6, equations (7) and (5) are plotted. It is worth noting that Wenzel and Cassie-Baxer models need to be considered together to completely describe the wettability of a surface^16^,^52^.

The one-dimensional Wenzel-Cassie-Baxter theory is not appropriate to describe the wetting properties of SWCNT films, since from equation (5) and Figure 6 it is clear that for a Young's contact angle *θ* ≈ 86° (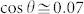) as for graphite, it is not possible obtaining any apparent contact angle θ* > 90° (cos θ* < 0). Furthermore, equation (5) implies that the maximum apparent contact angle θ* = 180° (cos θ* = −1) cannot be achieved for graphite, since *r* is defined as a positive quantity. Moreover, in equation (7) only one solid surface fraction *ϕ* with its own contact angle *θ* is present, while here we are dealing with a surface with at least two solid surface fractions with two different contact angles. This suggests that equations (5) and (7) need to be rearranged for a composite surface made of SWCNTs with two hierarchical roughness factors (*r_m_*, *r_n_*) and two corresponding solid fractions (*ϕ_m_*, *ϕ_n_*) with their respective Young's contact angles (*θ_m_*, *θ_n_*). There will also be the presence of an air (*ϕ*_−_) or liquid fraction (*ϕ*_+_), depending on the hydrophobic or hydrophilic state, respectively. If we assume at the first approximation, that both the two hierarchical roughness contribute to the surface wettability independently, by invoking the superposition principle we may write a two-dimensional extension of the Wenzel-Cassie-Baxter model as follows 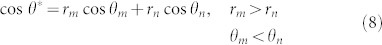

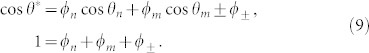


We reasonably assumed that for SWCNT film the micro-structure contact angle *θ_m_* = 86° as for graphite, therefore from equation (8) we obtained the nano-structure contact angle values 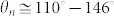 for our samples, as reported in Table 2. As expected, *θ_n_* is much higher than *θ_m_*, although the material is chemically the same. This is reasonable and can be ascribed to the hierarchical surface morphology. It has been already reported^12^,^53^ indeed, that the contact angle of a liquid droplet increases in the presence of two different scales of superimposed roughness, as in our case where micro-structures are made of nano-structures.

**Figure 7:**
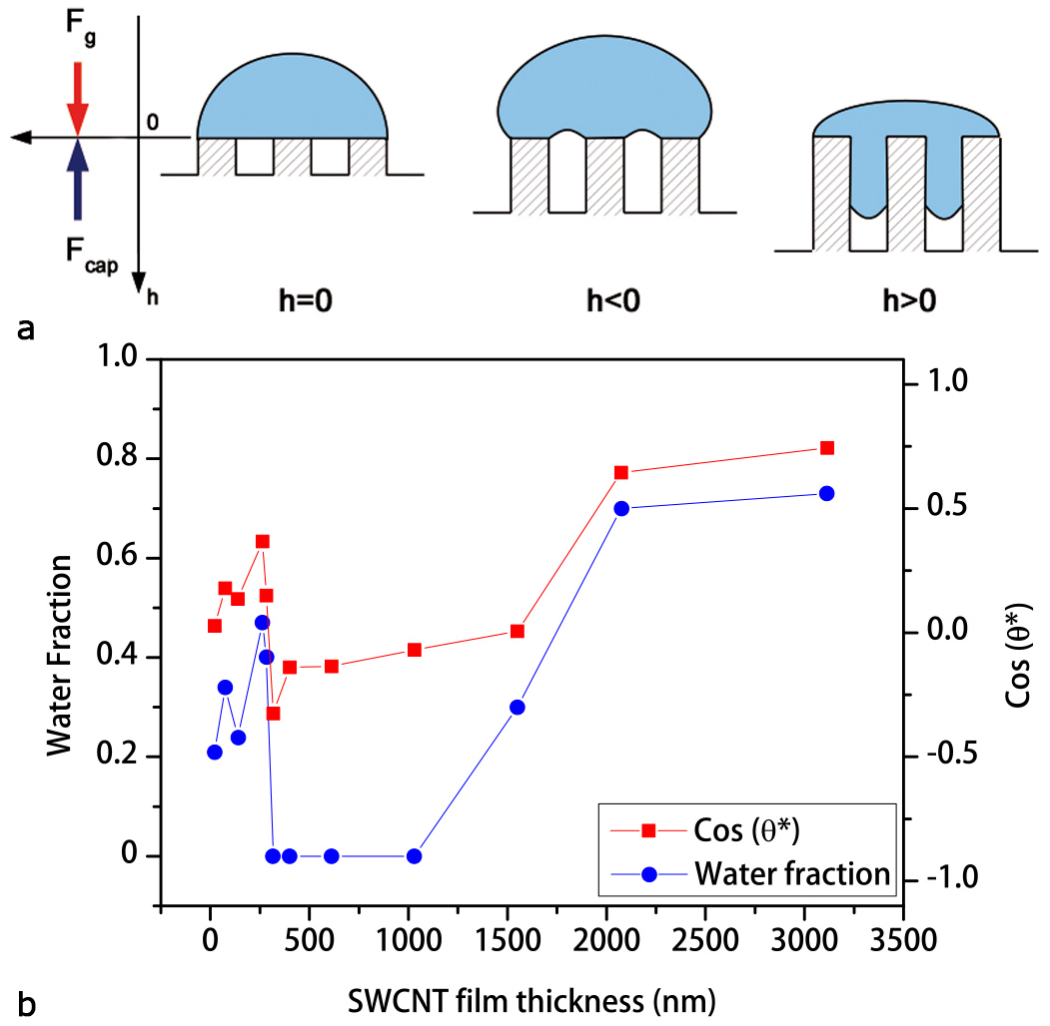
Capillary phenomena in SWCNT porous films. (a), Scheme of capillary phenomena in a generic porous media. (b), The plot shows cos θ* and the water fraction *ϕ*_+_ as functions of the SWCNT film thickness.

In addition, we computed with equation (9) numerical simulations of the surface fractions for our samples (see Table 2). The simulation consists in an algorithm, which for all the samples calculates cos θ* varying *ϕ_m_*, *ϕ_n_*, therefore *ϕ*_±_ by steps of 0.01. The resulting values of cos θ* are then compared with the experimental values 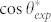 and minimized as follows 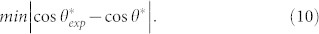
Interestingly, the micro- and nano-structure fractions do not depend on the apparent contact angle, therefore on film thickness. On the other hand, the water fractions follow the same trend of cos θ* as a function of the film thickness, as shown in Figure 7b. Therefore, we infer that the change in apparent contact angle with the coating thickness is strongly dependent on the water fraction. This suggests to interpret this behavior in terms of capillary phenomena.

### Capillary phenomena in single-walled carbon nanotube porous films

In general, on porous media the equilibrium contact angle can be defined only on average^15^. This is the consequence of the fact that porous media always have a random surface. In this situation, capillary rise (*i.e.* the liquid expulsion from pores) is likely to occur. A fundamental characteristic of porous media is the porosity Φ (*i.e.* the void fraction of the material total volume). It is now well-established that over certain length scales, pore surface and volume of most porous media are fractal^50^. Based on the aforementioned fractal picture, it has been suggested that the porosity of a medium may be written as^50^

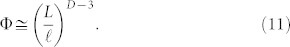
In Table 2 porosity values for our SWCNT films are reported with an average value Φ = 0.476 ± 0.001 over all samples, in agreement with the universal^50^ value for generic random networks.

Now we attempt to explain the variation of contact angle with the change in SWCNT film thickness *τ*, as reported in Figure 4a. Let us consider the system depicted in Figure 7a. A water drop sits on a porous SWCNT film, where the porosity Φ is due to the formation of a complex three-dimensional network of nano-channels into the coating. At first approximation, each nano-channel behaves as a vertical column developing a capillary force *F_cap_*, which at the equilibrium must balance the gravity force *F_g_*. Therefore, the equilibrium height *h* of the liquid column inside nano-channels can be written as^15^

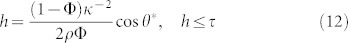
where *ρ* is the pore radius, θ* is the apparent contact angle, *τ* is the nano-channel length, and *κ*^−1^ = *ρτ* is the water capillary length in the nano-channels. Assuming the nano-channel length equivalent to the thickness of the SWCNT film, the capillary length in the coating results to be *κ*^−1^ ≈ 9 – 105 nm. In addition, since the equilibrium height *h* is proportional to the Laplace pressure *P* = *γ_LV_κ*^2^*h* (*i.e.* the change between the pressure outside and inside the pore), it is also proportional to the meniscus curvature *ρ*^−1^ = 2*P*/*γ_LV_*^15^. Therefore, when the equilibrium height between the capillary force *F_cap_* and the gravity force *F_g_* is at the pore aperture (*h* = 0 for *θ* = 90°), the liquid meniscus is flat; thus there is no water inside the capillary, nor air outside. On the other hand, for *θ* < 90°, *h* > 0 gravity force is stronger than capillary force, then water invades pores and the meniscus curvature *ρ*^−1^ is negative due to the negative Laplace pressure *P*. Conversely, for *θ* > 90°, *h* < 0 capillary force is higher than gravity force and the meniscus curvature is positive, because the positive pressure variation. This situation allows capillary rise, and eventually creating air pockets under the drop^54^.

In Figure 7b we observe that the equilibrium height *h* = 0 for *θ* = 90°, cos *θ* = 0 (flat meniscus condition) corresponds to a SWCNT film with a thickness *τ* ≈ 1550 nm. However, our simulation predicts a small water fraction *ϕ*_+_ ≈ 0.3%, which can be attributed to the metastable^54^ hydrophilic Wenzel-Cassie-Baxter state of the SWCNT film. For thicker coatings a water fraction permeates the pores leading to the hydrophilic state (for *θ* < 90°, cos *θ* > 0). This condition is further confirmed by our calculation, predicting a consistent water fraction (*ϕ*_+_ > 0.7) under the liquid droplet (Figure 7b). On the other hand, for thinner coatings (282 nm < *τ* < 1030 nm), the porous nature of the films coupled with strong capillary forces oppose water penetration, allowing atmosphere intake into the entangled nanotube network. Therefore, the hydrophobic state (for *θ* > 90°, cos *θ* < 0) is achieved, as shown in Figure 7b. Moreover, for *τ* < 282 nm pore length is so short that Laplace pressure is large and negative, thus capillary force is much weaker than gravity force and inevitably pores are fulfilled by water (for *θ* < 90°, cos *θ* > 0). This hypothesis is also confirmed by the presence of a fraction (*ϕ*_+_ = 0.21–0.47) of the droplet surface in contact with liquid, resulting from our calculations (Figure 7b). Capillary phenomena are hence testified by the presence of a water fraction varying accordingly with the SWCNT film thickness together with a change in hydrodynamic flow, as experimentally reported in Figure 4b.

In summary, we reported a promising approach to realize semi-transparent, hydrophobic and electrically conductive thin SWCNT coatings could be of main interest for inexpensive, fully scalable, highly reproducible, and effortless fabrication of future smart window applications^55^, photovoltaics^41^, and flexible electronics^56^. Furthermore, since SEM images of films revealed the hierarchical and multi-fractal nature of SWCNT random networks, we discussed the origin of the surface hierarchy, attributing it to a dry-induced self-assembly phenomenon. Also, roughness factors were extrapolated from the fractal analysis of SEM micrographs. Moreover, we suggested a two-dimensional extension of the Wenzel-Cassie-Baxter model to explain how the hierarchical micro- and nano-structured roughness play a crucial role leading to the hydrophobic regime. In addition, using our two-dimensional extension of the Wenzel-Cassie-Baxter model, we calculated by numerical simulations the water fraction present in the SWCNT porous films, showing their relation to capillary phenomena. In particular, SWCNT films of different thickness were prepared by vacuum filtration of an aqueous dispersion and deposited by dry-transfer printing at room temperature on several substrates, highlighting the versatility of this recent deposition technique. We then characterized optical, electrical, and wetting properties of as-realized films, showing that SWCNT coatings are highly conductive, broadband absorbers, and they result hydrophobic in the range of thickness ≈ (282–1030) nm. We also observed a remarkable change in wettability and in liquid suction process by varying the film thickness. Finally, future developments will aim to achieve higher contact angle values, by further lowering the surface tension of SWCNT composite surfaces by chemical treatments.

## Methods

### Fabrication of single-walled carbon nanotube films

Highly pure SWCNT powder (Sigma-Aldrich, carbon > 90%, SWCNTs > 77%, diameter: 0.7–0.9 nm) was dispersed in an aqueous solution (80 *µ*g mL^−1^) with 2% w/v sodium-dodecil-sulfate (Sigma-Aldrich, assay > 98.5%) anionic surfactant. Surfactant concentration (well above the critical micelle concentration ≈ 0.2%) was calibrated optimizing the carbon nanotube electrical response. In addition, to better disperse the suspension, SWCNTs were tip-ultrasonicated (Branson S250A, 200 W, 20% power, 20 KHz) in an ice-bath for an hour and the unbundled supernatant was collected by pipette. The result was a well-dispersed suspension, which is stable for several months. Single-walled carbon nanotube films were fabricated by a vacuum filtration process of aliquot volumes of the dispersion with mixed cellulose ester filters (Pall GN6, 1 in diameter, 0.45 *µ*m pore diameter). Subsequently, rinsing in water and in solution of ethanol, methanol and water (15:15:70) to remove as much surfactant as possible was performed.

### Sample preparation

Samples were made depositing by the dry-transfer printing method SWCNT films on several substrates such as Carlo Erba soda-lime glass slides, HF etched silicon (100) wafers, and plastic (cellulose acetate) foils. This recent deposition technique consists in soaking the SWCNT film with ethanol, in order to improve its adhesion, and then pressing it onto a substrate with a glass slide. After few minutes, the dried cellulose filter is removed by peeling it leaving the SWCNT film adhered on the substrate.

### Sample characterization

Optical spectroscopy (Perkin-Elmer Lambda 19 UV/Vis/NIR) was performed to characterize the thickness and the transparency of SWCNT films. Scanning electron microscopy micrographs were acquired with Zeiss Leo Supra 35 field emission scanning electron microscope (FEG-SEM) and analyzed with a threshold algorithm to measure pore and micro-structure distributions. Fractal analysis on SEM images was performed with the open-source software Gwyddion. Electrical measurements were conducted with a Keithley 2602A digital multimeter interfaced to a PC.

### Contact angle measurements

Images of sessile water drops cast on SWCNT films were acquired by a custom setup with a CCD camera. Static advanced contact angles were measured increasing the deionized water (18.2 MΩ cm) drop volume by step of 1 *µ*L obtaining the maximum value for *V* = 10 *µ*L. Furthermore, every contact angle was measured 15 s after drop casting to ensure that the droplet reached its equilibrium position. Moreover, a plugin^57^ for the open-source software ImageJ was exploited to estimate the contact angle values. This plugin uses an algorithm based on a small-perturbation solution of the Young-Laplace equation^14^. Furthermore, this method is applied to a continuous image of the droplet, by using cubic B-Spline interpolation of the drop contour to reach subpixel resolution, with an accuracy of 0.02°.

### Fractal analysis

The fractal analysis was carried out with the open-source software Gwyddion. The triangulation method works as follows: a lattice of unit dimension *x* is placed on the surface SEM image. This defines the location of the vertexes of a number of triangles. Initially *x* is set at 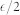 (where 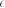 is length of edge of the image), then the surface is covered by 8 triangles of different areas inclined at various angles with respect to the plane. Hence, *N*(*x*) is the number of all triangles that contain at least one pixel of the image, therefore obtaining an approximation of the surface area of the fractal. The lattice size is then decreased stepwise by a factor of 2 and the process continues until *x* corresponds to the distance between two adjacent pixels of the image. Since *N*(*x*) = *x*^−*δ*^, from the slope of log *N*(*x*)-log *x* plot the fractal dimension *D* = *δ* + 2 may be obtained.

## Author Contributions

F.D.N., P.C., M.S., I.C., F.N. and M.D.C. conceived the experimental approach for the contact angle measurement on SWCNT coatings. F.D.N. fabricated SWCNT films, performed their optical and electrical characterization, their fractal analysis, and carried out the contact angle experiments. I.C. and F.N. acquired SEM images of the coatings. F.D.N. and P.C. contributed to the theoretical analysis on the two-dimensional Wenzel-Cassie-Baxter model. All authors discussed the experimental implementation and results and contributed to writing the paper.

## Supplementary Material

Supplementary Information Supplementary Informations

## Figures and Tables

**Figure 1:**
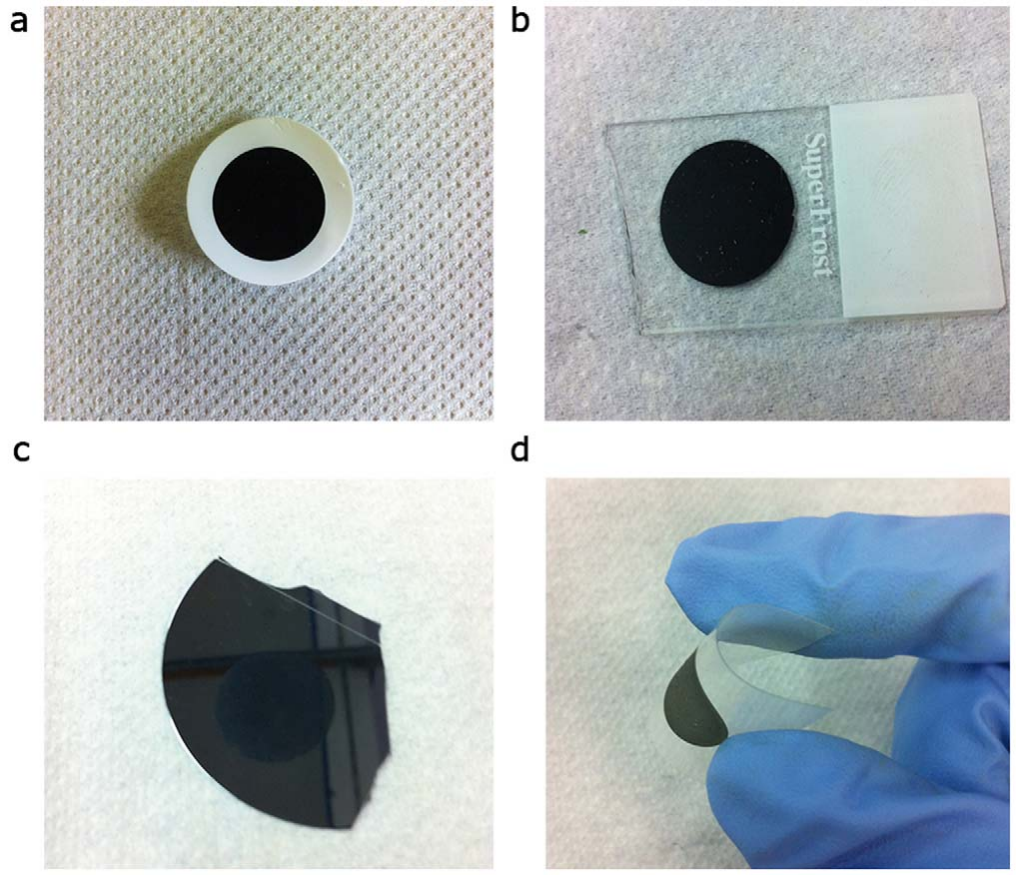
Versatility of the dry-transfer printing method. A SWCNT film realized on cellulose filter (a) may be deposited on glass (b), silicon (c), and plastic foils (d).

**Table 1:** Experimental results of contact angle measurements and fractal analysis. Uncertainties on fractal dimensions are systematic errors, otherwise propagated errors

Sample	Thickness (nm)	Contact angle (deg)	*D_m_*	*D_n_*	*r_m_*	*r_n_*	Φ
Glass	-	33.4	-	-	-	-	-
Silicon	-	74.0	-	-	-	-	-
Plastics	-	70.3	-	-	-	-	-
1 SWCNT	22 ± 3	88.4	2.85 ± 0.03	2.44 ± 0.03	18.87 ± 0.01	1.844 ± 0.006	0.461 ± 0.002
2 SWCNT	74 ± 9	79.7	2.85 ± 0.03	2.48 ± 0.03	19.27 ± 0.02	1.943 ± 0.006	0.486 ± 0.002
3 SWCNT	138 ± 18	82.2	2.87 ± 0.03	2.47 ± 0.03	20.79 ± 0.02	1.926 ± 0.007	0.481 ± 0.002
4 SWCNT	317 ± 41	68.5	2.84 ± 0.03	2.43 ± 0.03	18.76 ± 0.01	1.827 ± 0.006	0.457 ± 0.002
5 SWCNT	264 ± 34	81.4	2.77 ± 0.03	2.40 ± 0.03	14.42 ± 0.01	1.736 ± 0.005	0.434 ± 0.002
6 SWCNT	282 ± 36	109.4	2.87 ± 0.03	2.42 ± 0.03	20.74 ± 0.02	1.785 ± 0.006	0.446 ± 0.002
7 SWCNT	400 ± 51	98.0	2.85 ± 0.03	2.56 ± 0.03	19.35 ± 0.02	2.176 ± 0.009	0.543 ± 0.001
8 SWCNT	612 ± 32	97.8	2.86 ± 0.03	2.50 ± 0.03	19.69 ± 0.02	1.998 ± 0.008	0.499 ± 0.002
9 SWCNT	1030 ± 52	94.0	2.81 ± 0.03	2.49 ± 0.03	16.72 ± 0.01	1.971 ± 0.008	0.492 ± 0.002
10 SWCNT	1550 ± 83	89.7	2.77 ± 0.03	2.43 ± 0.03	14.50 ± 0.01	1.813 ± 0.006	0.453 ± 0.002
11 SWCNT	2074 ± 104	49.9	2.85 ± 0.03	2.50 ± 0.03	18.87 ± 0.01	2.004 ± 0.008	0.501 ± 0.002
12 SWCNT	3114 ± 16	42.0	2.76 ± 0.03	2.44 ± 0.03	14.03 ± 0.01	1.865 ± 0.007	0.466 ± 0.002

**Table 2:** Results of numerical simulations

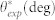	*θ_m_* (deg)	*θ_n_* (deg)	*ϕ_m_*	*ϕ_n_*	*ϕ*_+_	*ϕ*_−_
88.4	86.0	134.3	0.19	0.60	0.21	-
79.7	86.0	126.8	0.35	0.31	0.34	-
82.2	86.0	132.8	0.55	0.21	0.24	-
68.5	86.0	121.0	0.29	0.24	0.47	-
81.4	86.0	119.5	0.08	0.52	0.40	-
109.4	86.0	175.4	0.62	0.38	-	-
98.0	86.0	133.2	0.79	0.05	-	0.16
97.8	86.0	139.1	0.76	0.21	-	0.03
94.0	86.0	128.8	0.80	0.20	-	-
89.7	86.0	123.7	0.15	0.55	0.30	-
49.9	86.0	109.6	0.11	0.19	0.70	-
42.0	86.0	97.2	0.24	0.03	0.73	-
