# Supplementary material for: Multi-Fractal Hierarchy of Single-Walled Carbon Nanotube Hydrophobic Coatings
Source: Sci Rep. 2015 Feb 26;5:8583. doi: 10.1038/srep08583 (PMC4341200; doi:10.1038/srep08583)
Supplement: Supplementary Information — s [file srep08583-s1.pdf]

Supplementary Informations to Multi-Fractal Hierarchy of Single-Walled Carbon Nanotube Hydrophobic Coatings

Francesco De Nicola,[1,2,*] Paola Castrucci,[1,2] Manuela Scarselli,[1,2]
Francesca Nanni,[3] Ilaria Cacciotti,[4] and Maurizio De Crescenzi[1,2,5]

[1]*Dipartimento di Fisica, Universitá di Roma Tor Vergata, Via della Ricerca Scientifica 1, 00133 Roma, Italy*

[2]*Istituto Nazionale di Fisica Nucleare, Universitá di Roma Tor Vergata (INFN-Roma Tor Vergata), Via della Ricerca Scientifica 1, 00133 Roma, Italy*

[3]*Dipartimento di Ingegneria dell'Impresa, Universitá di Roma Tor Vergata (INSTM-UdR Roma Tor Vergata), Via del Politecnico 1, 00133 Roma, Italy*

[4]*Universitá di Roma Niccoló Cusano (INSTM-UdR), Via Don Carlo Gnocchi 3, 00166 Roma, Italy*

[5]*Istituto di Struttura della Materia, Consiglio Nazionale delle Ricerche (ISM-CNR), Via del Fosso del Cavaliere 100, 00100 Roma, Italy*

(Dated: February 2, 2015)

* Correspondence to fdenicola@roma2.infn.it

I. HIERARCHICAL SELF-ASSEMBLY MORPHOLOGY OF SINGLE-WALLED CARBON NANOTUBE FILMS

The scanning electron microscopy (SEM) images of our single-walled carbon nanotube (SWCNT) films reported in the main text (Figure 2a-d), clearly show the presence of micrometric ripples, emerging from the film surface. In particular, it is clearly visible by inspecting SEM images at higher magnifications, that this micro-structured morphology is made of self-assembly SWCNT bundles. These features are typical of every SWCNT films obtained by our fabrication process described in the main text (see Methods). In the following, we suggest a mechanism driving the micro-structured morphology self-assembly.

It has been already reported that the competition between capillary forces arising during the evaporation of liquid solvents from dense carbon nanotube arrays and bending stress due to the elasticity of carbon nanotubes provide the self-assembly of vertical aligned carbon nanotubes into two-dimensional contiguous cellular foams[1,2]. In our case, the observed hierarchical self-assembly morphology of our SWCNT coatings is due to a bending process during evaporative drying of the film, once deposited on a substrate. Therefore, in the nanoporous film a dry-induced self-assembly occurs as a consequence of the attractive capillary force, together with convective flows of the evaporating liquid solvent. The result is an out-of-plane deformation of the material, causing film bending and aggregation of nanotube bundles. We estimated a maximum capillary force $F_{cap} \approx 0.3$ nN in the porous films by the following relation[3]

$$F_{cap} = \frac{S\gamma_{LV}\,(1-\Phi)}{2\rho}\cos\theta^*, \tag{1}$$

where $S \approx 71$ nm^2 is the pore area, $\Phi \approx 0.50$ the film porosity, $\rho \approx 4.3$ nm the pore radius, $\gamma_{LV} = 72$ mJ m^{-2} the water surface tension[3], and θ^* the apparent contact angle between the liquid solvent and the solid surface. On the other hand, the dry-induced bending stiffness of the film must balance the capillary force. Since our SWCNT thin film is a circular membrane, according to the Kirchoff-Love plate theory[4] for an isotropic, homogeneous, thin, and circular plate the maximum bending stiffness Δ is given by

$$\Delta = EI = \frac{\tau^3 E}{12\,(1-\nu^2)}, \tag{2}$$

where E is the carbon nanotube film elastic or Young's modulus, $I = \tau^3/12$ is the second moment of area of an isotropic and homogeneous thin plate, ν is the Poisson's ratio, and τ

is the film thickness. For instance, for a carbon nanotube film ≈ 3 μm thick, a Poisson's ratio $\nu \approx 0.3$[5], and an elastic modulus $E \approx 10$ MPa[6], we found $\Delta \approx 28$ pJ. Then, we can write the first order approximation at Δ of the maximum mid-surface out-of-plane radial deflection of a constant and symmetrically forced plate writes[4]

$$\omega(r) \approx \frac{r^4 F_{cap}}{64 \Sigma \Delta}, \tag{3}$$

being $r = 0.75$ cm the radius and $\Sigma = 1.77$ cm^2 the area of the film. The estimated maximum out-of-plane mid-plate radial deflection $\omega(r) \approx 3$ μm is thus comparable with the micro-structure height $\xi \approx 3$ μm of our SWCNT film.

II. FRACTAL ANALYSIS

There are several techniques for practical estimation of fractal dimension of a real surface, such as the box or cube counting method,[7] the triangulation algorithm,[7] the variogram,[8] and the Fourier power spectrum analysis.[7]

In the box counting algorithm[7] (see Figure 1a) a square lattice with lattice constant x is superimposed on the surface SEM image. Initially x is set at $\epsilon/2$ (where ϵ is length of edge of the surface image), resulting in a lattice of $2^2 = 4$ boxes. Then $N(x)$ is the number of all squares that contain at least one pixel of the image. The lattice constant x is then reduced stepwise by factor of 2 and the process repeated until x equals to the distance between two adjacent pixels. The slope of the $\log N(x)$-$\log x$ plot gives the fractal dimension D directly.

The triangulation method[7] (see Figure 1b) is very similar to cube counting method and is also based directly on the box-counting fractal dimension definition. The method works as follows: a lattice of unit dimension x is placed on the surface SEM image. This defines the location of the vertexes of a number of triangles. Initially x is set at $\epsilon/2$ (where ϵ is length of edge of the image), then the surface is covered by 8 triangles of different areas inclined at various angles with respect to the plane. Hence, $N(x)$ is the number of all triangles that contain at least one pixel of the image, therefore obtaining an approximation of the surface area of the fractal. The lattice size is then decreased stepwise by a factor of 2 and the process continues until x corresponds to the distance between two adjacent pixels of the image. Since $N(x) = x^{-\delta}$, from the slope of $\log N(x)$-$\log x$ plot the fractal dimension $D = \delta + 2$ may be obtained.

The variogram[8] (see Figure 1c) is based on the scale dependence of the variance of the fractal surface. In practice, the variogram consists in dividing the full surface of the SEM image into equal-sized squared boxes, and the variance of the gray-scale values of the height profiles is calculated for a particular box size. Since $V(x) \propto x^{-\beta}$, the fractal dimension is evaluated from the slope β of a least-square regression line fit to the data points in $\log V(x)$-$\log x$ plot of variance as $D = 3 - \beta/2$.

The Fourier power spectrum analysis[7] (see Figure 1d) is based on the power spectrum dependence of the fractal surface. In this method, each of the 1024 line height profiles that form the SEM image (each consisting of 768 points with a gray-scale value) is Fourier transformed and the power spectrum evaluated. The power spectra $F(k) \propto k^{\eta}$ are averaged and the average integrated from high to low wavelength. Therefore, fractal dimension is evaluated from the slope η of a least-square regression line fit to the data points in $\log N(x)$-$\log x$ plot of power spectrum as $D = 7/2 - \eta/2$.

Furthermore, we established the validity of all those algorithms testing them on deterministic fractals (*i.e.* Sierpinski triangle, Sierpinski carpet, Koch curve, *etc.*) with known fractal dimensions D_{theo}, as plotted in Figure 1e. We found that not only the triangulation method is one of the most reliable algorithms to estimate the fractal dimension of a surface but also it is sensitive to the multi-fractality of our SWCNT films, as shown in Figure 1b.

Moreover, we performed the same fractal analysis at different magnification or pixel densities of the SEM images. Figure 1f shows that fractal lengths (*i.e.* the correlation length within the fractal law holds) shift with the image density of pixels, accordingly to the scale of the structures depicted in the SEM image.

In order to estimate the systematic error on the fractal dimensions we averaged the semi-dispersion between the known theoretical values of the test fractals and the measured values, over all the tested fractals.

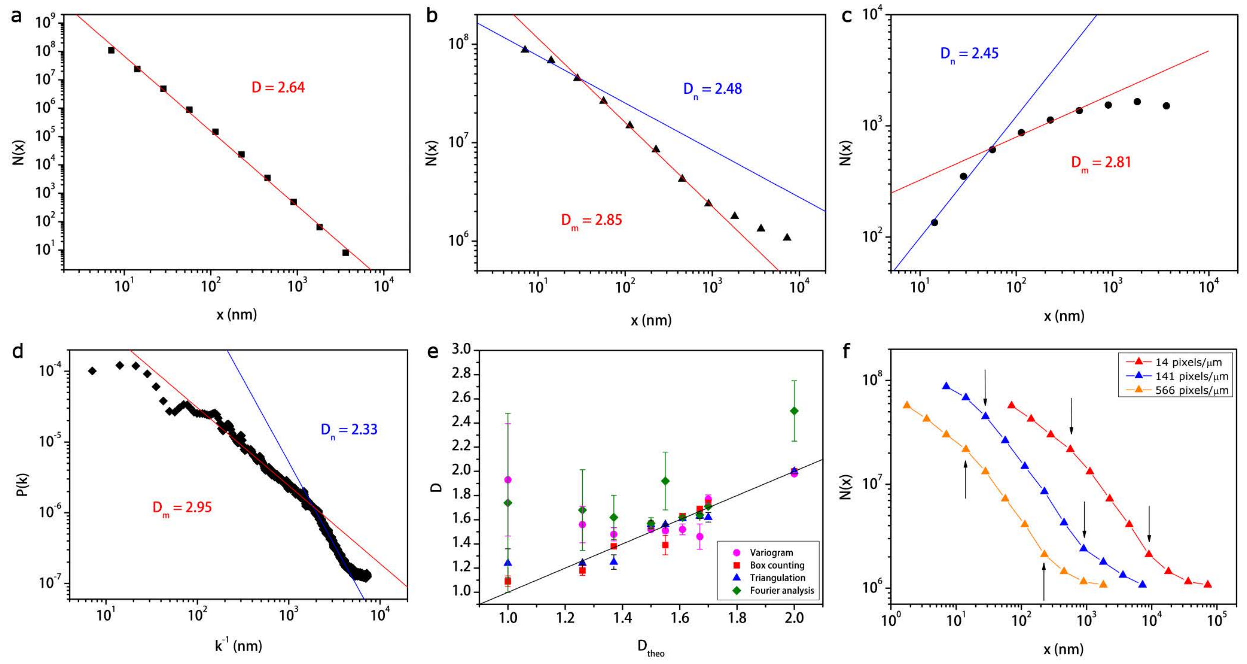

FIG. 1. Fractal analysis of a SWCNT film. **a**, We obtained by the box counting method a fractal dimension $D = 2.64 \pm 0.03$. **b**, The triangulation method distinguishes two fractal dimensions relative to the hierarchical morphology $D_n = 2.48 \pm 0.03$ and $D_m = 2.85 \pm 0.03$. **c**, Also the variogram estimated two fractal dimensions $D_n = 2.45 \pm 0.03$ and $D_m = 2.81 \pm 0.03$. **d**, We had by Fourier analysis the two fractal dimensions $D_n = 2.33 \pm 0.03$ and $D_m = 2.95 \pm 0.03$. Furthermore, the Fourier power spectrum shows peaks corresponding to a periodic pattern. This feature is characteristic of a structured morphology. **e**, We established the validity of those algorithms testing them with deterministic fractals with known fractal dimensions. The triangulation method (blue triangles) and the box counting method (red squares) fit the linear relation (black solid line) between the known fractal dimensions D_{theo} and the measured D better than the variogram (magenta dots) and the Fourier analysis (green diamonds). **f**, The maximum and minimum micro-structure fractal lengths (black arrows) in $\log N(x) - \log x$ plots shift with image density of pixels reflecting the fact that in SEM images at low (high) magnification only the micro-structures (nano-structures) are present. The estimation of the systematic error on D is the average of the semi-dispersion between the known theoretical values of the test fractals and the measured values, over all the tested fractals.

[1] Li, Q. et al. Drying induced upright sliding and reorganization of carbon nanotube arrays. *Nanotechnology* **17**, 4533–4536 (2006).

[2] Chakrapani, N., Wei, B., Carrillo, A., Ajayan, P. M. & Kane, R. S. Capillarity-driven assembly of two-dimensional cellular carbon nanotube foams. *PNAS* **101**, 4009–4012 (2004).

[3] De Gennes, P.-G., Brochard-Wyart, F. & Quéré, D. *Capillarity and wetting phenomena* (Springer, New York, 2003).

[4] Reddy, J. N. *Theory and Analysis of Elastic Plates and Shells* (CRC Press, Boca Raton, 2007).

[5] Zhao, P. & Shi, G. Study of poisson's ratios of graphene and single-walled carbon nanotubes based on an improved molecular structural mechanics model. *S. L.* **5**, 49–58 (2011).

[6] Olofsson, N., Ek-Weis, J., Eriksson, A., Idda, T. & Campbell, E. E. B. Determination of the efeffect young's modulus of vertically aligned carbon nanotube arrays: a simple nanotube-based varactor. *Nanotechnology* **20**, 385710–385716 (2009).

[7] Douketis, C., Wang, Z., Haslett, T. L. & Moskovits, M. Fractal character of cold-deposited silver films determined by low-temperature scanning tunneling microscopy. *Phys. Rev. B* **51**, 11022–11031 (1995).

[8] Van Put, A., Vertes, A., Wegrzynek, D., Treiger, B. & Van Grieken, R. Quantitative characterization of individual particle surfaces by fractal analysis of scanning electron microscope images. *Fresen. J. Anal. Chem.* **350**, 440–447 (1994).
